# Managing BMI and Emotional Distress Using mHealth: Nationally Representative Survey Study

**DOI:** 10.2196/75499

**Published:** 2026-06-03

**Authors:** Ranran Z Mi, Fei Shen, Julia Strugala

**Affiliations:** 1 Department of Communication, Media and Journalism, Kean University, 1000 Morris Ave, Union, NJ, 07083, United States, 1 908-737-0456, 1 908-737-0465; 2 School of Psychology, Kean University, Union, NJ, United States

**Keywords:** mobile health, wearable trackers, eating, dietary, physical activity, sleep, emotional distress, body mass index

## Abstract

**Background:**

Mobile health (mHealth) technologies, including smartphone health apps and wearable trackers, are increasingly used to promote health behaviors. However, their impact on physical and mental well-being remains complex, with both benefits and potential unintended negative consequences.

**Objective:**

This study aimed to examine the relationship between mHealth use (ie, health app and wearable tracker) and 2 health outcomes (BMI and emotional distress), as well as the mediating roles of healthy eating, sleep, and physical activity based on a representative sample.

**Methods:**

We analyzed data from a nationally representative sample of US adults aged 33 to 43 years (N=1931). Chi-square tests and 1-way ANOVA were used to compare demographic differences between mHealth users and nonusers. A path model examined the relationship between mHealth use (ie, smartphone health apps and wearable trackers) and health outcomes (ie, BMI and emotional distress), with lifestyle factors (ie, healthy eating, physical activity, and sleep) as mediators. Mediation analyses tested indirect effects through these lifestyle factors.

**Results:**

mHealth users were more likely to be female, married, have higher levels of education and income, and have health insurance. The primary use of mHealth was the management of physical activity. Smartphone health app use positively correlated with wearable tracker use (β=.394; *P*<.001). Smartphone health app use predicted greater BMI (β=.068; *P*=.006), whereas wearable tracker use did not significantly predict BMI. Smartphone health app use was unrelated to emotional distress, while wearable tracker use was associated with lower emotional distress (β=–.074; *P*=.003). Mediation analyses showed that physical activity negatively mediated the relationships between both types of mHealth use and health outcomes, indicating that mHealth users were more physically active, which was linked to lower BMI and emotional distress. Sleep hours mediated only the association between wearable tracker use and health outcomes, such that greater tracker use was related to fewer sleep hours, predicting higher BMI and emotional distress. Finally, healthy eating mediated only the associations between mHealth use and emotional distress, suggesting that healthier dietary behaviors among mHealth users contributed to lower emotional distress.

**Conclusions:**

mHealth technologies can potentially promote healthier behaviors, but their effectiveness depends on users taking the initiative to sustain lifestyle changes. While wearable trackers may aid in mental well-being, their association with reduced sleep warrants further investigation.

## Introduction

### Background

Mobile health (mHealth) technologies, including smartphone apps and wearable trackers, have become increasingly popular tools for managing personal health and wellness. Approximately half of the US population has at least 1 mHealth app on their mobile devices [1], and 21% of US adults regularly use a wearable tracker or smartwatch, a number that has increased due to the COVID-19 pandemic and the subsequently growing fitness culture [2]. While previous studies have highlighted socioeconomic disparities in mHealth adoption [3], these technologies continue to expand in popularity due to their convenience, personalized nature, and growing integration with health care systems [4,5]. Research increasingly supports mHealth’s potential for managing health outcomes, such as weight management [6] and emotional regulation [7].

Despite the growing body of evidence, a significant gap remains: studies that aim to assess the effectiveness of mHealth technology either focus on behavioral lifestyle as the end outcome or examine physical or mental health outcomes without exploring the underlying mechanism. It is critical to empirically investigate whether short-term behavioral changes induced by mHealth technology are linked to long-term health outcomes. Therefore, a more comprehensive analysis is needed to clarify the direct and indirect associations between mHealth use and health outcomes. By examining lifestyle factors such as diet, sleep, and physical activity as mediators, this study aims to provide deeper insights into the potential mechanisms driving these relationships. On the basis of a nationally representative sample of middle-aged US adults, this study explores the relationships between mHealth use (ie, health apps and wearables) and both physical and mental health outcomes (ie, BMI and emotional distress), while also examining the mediating roles of lifestyle factors (ie, diet, sleep, and physical activity).

### mHealth Technology Use

Smartphone health apps and wearable trackers have seen increased adoption, with distinct demographic patterns among their users. Research shows that age and socioeconomic status play a critical role in mHealth adoption rates. For instance, these technologies are predominantly used by younger individuals with higher levels of education and income who come from more affluent households [8]. A gender difference also stands out in mHealth use, as research has found that women are 2.3 times more likely to use wearable health trackers, a trend further substantiated by data from the Pew Research Center [2,9].

Over a decade of research has reported that health apps and wearable trackers are used for various health purposes such as weight management, stress coping, substance use disorder recovery support, chronic condition monitoring, telemedicine support, and electronic medical records [10-13]. The types of health aspects managed through these technologies are expanding to lifestyle domains, including documenting dietary habits, monitoring physical activity, and tracking sleep patterns [14,15]. By providing real-time feedback, mHealth tools encourage users to reflect on their behaviors and make informed decisions, leading to better management of various health aspects.

### mHealth and Weight Management

Weight management is a critical focus of mHealth technologies due to its profound implications for physical well-being. A systematic review found that mHealth technologies had a small but statistically significant effect on BMI reduction in children and adolescents [16]. While research supports the association between mHealth technology use and weight management, this relationship is likely mediated by lifestyle factors such as eating habits [17], sleep patterns [18], and physical activities [19,20]. For instance, app use is associated with healthy eating motives, decisions, and behaviors [21,22]. Wearable technologies further enhance self-monitoring by automatically detecting eating behaviors and tracking dietary intake [23,24]. Additionally, mHealth tools help reduce sedentary behavior and encourage physical activity [9]. A meta-analysis found that the use of activity trackers was associated with an average daily increase of 1800 steps, 40 minutes of walking, and 1 kg weight loss [25]. Sleep tracking is also designed as a key feature of mobile devices, aiming to improve sleep quality. Sleep-tracking features in mHealth tools enable users to identify patterns, address sleep issues, and access actionable insights that improve sleep and related health outcomes [26,27]. Evidence, although limited, shows wearable tracker use improves sleep quality among the health population [28].

### mHealth and Emotional Distress

Previous research has shown that mHealth technologies can monitor mental health and alleviate emotional distress [29,30], with users reporting a greater sense of responsibility for their well-being and confidence in the accuracy of app-generated assessments [31]. Additional studies have suggested that engagement with mood-tracking apps can potentially improve users’ ability to cope with unpleasant emotions and thoughts [32].

Additional studies suggest that lifestyle factors, especially physical activity and dietary habits, mediate these effects [33-36]. For example, a US national survey found that wearable users reported lower psychological distress, attributing this to longer workouts [37]. A randomized trial found that mindfulness app users, compared to nonusers, reported reduced emotional eating and stress levels, suggesting mHealth contributes to stress reduction by reducing emotional eating [38]. Additionally, mobile devices can track meal timing, location, and social context to encourage communal eating and reduce skipping meals, which correlates with elevated stress, anxiety, and depression [39]. Research on the mediating role of sleep in the relationship between mHealth use and emotional well-being remains limited. While one study suggests that sleep app use is positively associated with perceived well-being [40], it remains unclear whether actual sleep serves as a mediating factor in this process.

Taken together, the literature provides consistent evidence for meaningful relationships between mHealth and health (ie, BMI and emotional distress), as well as how lifestyle behaviors might play roles in such relationships. Detangling the lifestyle mechanisms will inform strategies for optimizing mHealth tools to improve overall health and guide future interventions. In tackling the gaps, we posed four hypotheses (H): (1) H1: the use of (a) smartphone health apps and (b) wearable trackers is associated with lower BMI; (2) H2: the use of (a) smartphone health apps and (b) wearable trackers is associated with lower emotional distress; (3) H3: healthy eating, sleep hours, and physical activities mediate the relationships between (a) the use of smartphone health apps and BMI and (b) the use of wearable trackers and BMI; and (4) H4: healthy eating, sleep hours, and physical activities mediate the relationships between (a) the use of smartphone health apps and emotional distress and (b) the use of wearable trackers and emotional distress.

## Methods

### Participants

Data were retrieved from the National Longitudinal Study of Adolescent to Adult Health (Add Health), a 5-wave nationally representative sample of adolescents’ health and health-related behaviors in the United States. Add Health uses a complex, stratified, and cluster sampling design [41]. More than 20,000 adolescents in grades 7 to 12 (aged 12‐19 years) from more than 80 high schools and 52 feeder middle schools were interviewed at school and home in wave I (1994-1995). The participants’ backgrounds were diverse in terms of locations, school types, regions, and ethnicities. Participants were followed up in 1996 (wave II; aged 13‐20 years), 2001 to 2002 (wave III; aged 18‐26 years), 2008 (wave IV; aged 24‐32 years), and 2016 to 2018 (wave V; aged 33‐43 years).

### Ethical Considerations

Permission to conduct a secondary analysis was approved by Kean University’s Institutional Review Board. The original consent and institutional review board authorization included provisions for secondary data analyses; thus, additional consent was not required. In this study, we conducted analyses using the deidentified public-use data from wave V of the full Add Health sample (2016‐2018). The final analytic sample consisted of 1931 participants who provided valid responses to health technology questions.

### Measures

#### Smartphone Health App Use

Participants indicated whether they used smartphone apps to track or manage their health. Responses were coded as 1 for “yes” and 0 for “no.”

#### Wearable Tracker Use

Participants reported whether they had ever used a fitness or activity tracker. Responses were coded as 1 for “yes” and 0 for “no.”

#### Healthy Eating

Participants reported the frequency of (1) consuming food from fast-food restaurants and (2) drinking nondiet sweetened beverages over the past 7 days. The frequencies were summed and reverse-coded so that higher scores indicated healthier eating behaviors.

#### Sleep Hours

Participants reported their average number of sleep hours per day/night.

#### Physical Activity

Participants reported how often they engaged in various physical activities (eg, biking, weightlifting, swimming, and golfing) over the past 7 days. These frequencies were summed to create a composite physical activity score such that higher scores indicate a higher level of physical activity.

#### BMI

BMI was calculated based on participants’ self-reported height and weight, following the formula BMI=weight (in kilograms)/height (in meters)^2^ [42].

#### Emotional Distress

Emotional distress was measured using a set of items assessing participants’ feelings over the past 7 days. Participants indicated how often each of the following statements was true for them on a 4-point Likert scale: (1) “I felt that I could not shake off the blues, even with help from my family and friends,” (2) “I felt depressed,” (3) “I was happy” (reverse-coded), (4) “I felt sad,” and (5) “I felt that life was not worth living.” Responses were scored on a scale ranging from 1=“Never or rarely” to 4=“Most of the time or all of the time.” A composite score was calculated, with higher scores indicating greater emotional distress (α=.82).

#### Covariates

Participants’ age at survey completion, biological sex, education, household income, race, and ethnicity were included as covariates in all analyses.

### Analytic Approach

We used R (R Foundation for Statistical Computing) to perform all analyses. The “tableone” package was used to provide descriptive statistics and perform statistical comparisons between users and nonusers of mHealth [43]. More specifically, chi-square tests were performed on categorical variables, and 1-way ANOVA was used for continuous variables to compare differences.

**Figure 1.**
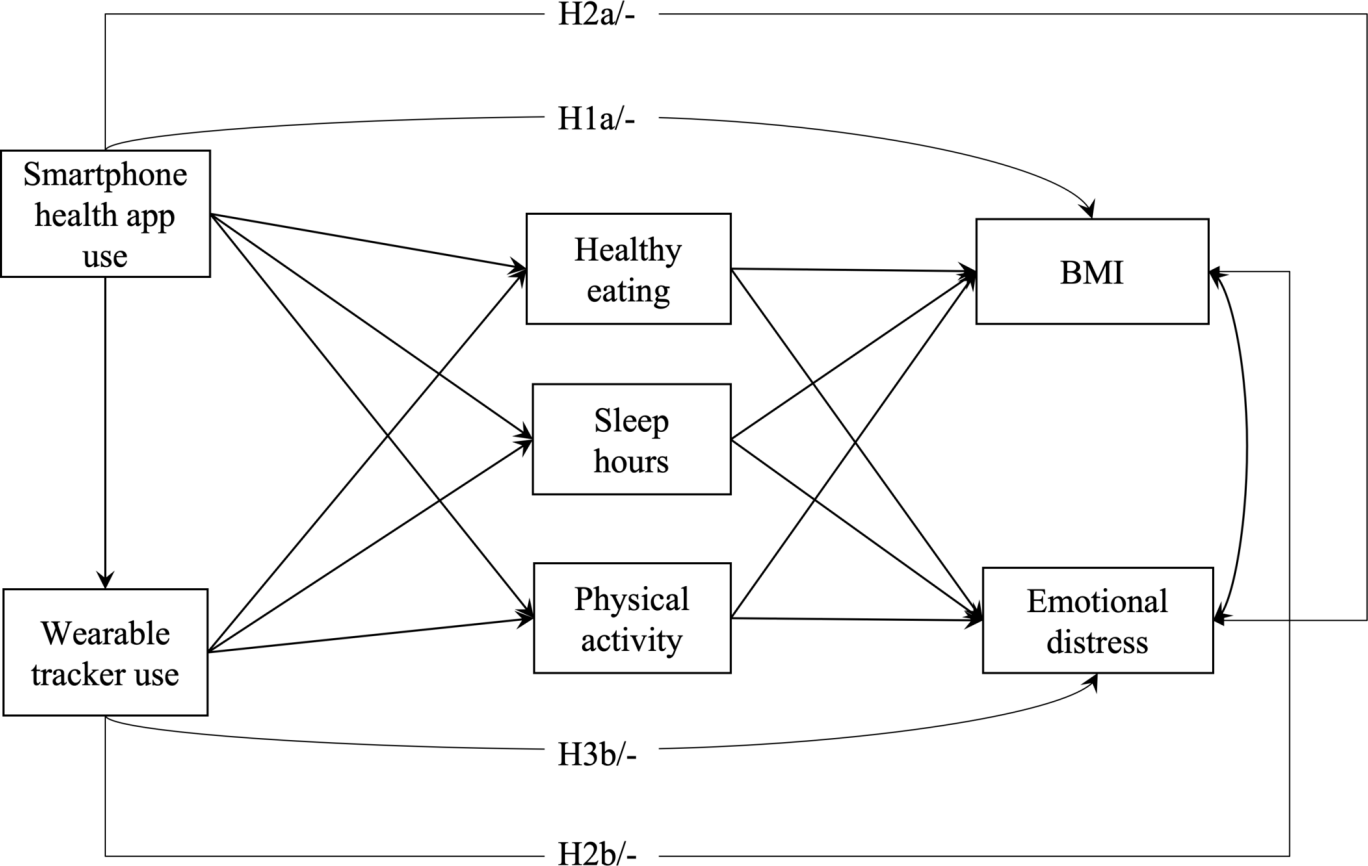
Hypothesized paths testing the relationship among mobile technology use, lifestyle mediators, and health outcomes. Note: H1: the use of (a) smartphone health apps and (b) wearable trackers is associated with lower BMI; H2: the use of (a) smartphone health apps and (b) wearable trackers is associated with lower emotional distress; H3: healthy eating, sleep hours, and physical activities mediate the relationships between (a) the use of smartphone health apps and BMI and (b) the use of wearable trackers and BMI; H4: healthy eating, sleep hours, and physical activities mediate the relationships between (a) the use of smartphone health apps and emotional distress and (b) the use of wearable trackers and emotional distress. H3 and H4 hypothesize that healthy eating, sleep hours, and physical activity mediate the relationship between mobile health use (smartphone health app use and wearable tracker use) and health outcomes (BMI and emotional distress).

To test the hypotheses, path analysis was performed using “lavaan” package [44]. The hypothesized paths are depicted in Figure 1. In the model, mobile technology use variables (ie, smartphone health app use and wearable tracker use) were specified as predictors of health outcomes (ie, BMI and emotional distress), with 3 lifestyle variables (ie, healthy eating, sleep hours, and physical activity) included as mediators. Mediation analyses were conducted to assess the indirect effects of mobile technology use on health outcomes through lifestyle mediators. All path coefficients were estimated using maximum likelihood with robust SEs to address nonnormality, and full information maximum likelihood estimation was used to handle missing data. Indirect effects were assessed using bias-corrected bootstrapped 95% CIs based on 5000 resamples. Age, sex, education, household income, race, and ethnicity were controlled for as covariates.

As the public-use version of Add Health Wave V does not include design variables (weights, primary sampling units, or strata), all analyses were conducted without applying survey design variables. Given that the primary objective was to examine internal associations rather than to produce nationally representative estimates, unweighted path analyses were deemed appropriate, and the findings should be interpreted accordingly [45].

## Results

### Descriptives

Our analysis, based on a nationally representative sample, revealed distinct demographic trends among smartphone health app users and wearable tracker users. Specifically, smartphone health app users and wearable tracker users share the following characteristics in common: they are more likely to be female, married, have higher levels of education and income, and possess health insurance, compared with their nonuser counterparts. In addition, smartphone health app users are more likely to be younger, identify as liberal, and have 1 or no children compared to nonusers. Wearable tracker users are more likely to own the place where they live compared to their nonuser counterparts. Detailed participants’ characteristics can be found in Table 1.

Participants use smartphones to manage a range of personal health aspects, with physical activity (714/842, 84.8%) being the most common, followed by diet management (399/838, 47.6%) and other aspects (Table 2). Female users (534/851, 62.7%) also reported using smartphones for menstrual cycle tracking (182/534, 34.1%) and pregnancy tracking (57/534, 11%). See Table 2 for the full list of health management aspects. Descriptive statistics and a correlation matrix for endogenous variables in the path model were reported in Table 3.

**Table 1.** Participant characteristics (N=1931)^a^.

	Overall	Smartphone health app use			Wearable tracker use		
		No (n=1080)	Yes (n=851)	*P* value	No (n=1251)	Yes (n=665)	*P* value
Sex, n (%)				<.001^b^			.001^c^
Female	1106 (57.3)	572 (53.0)	534 (62.7)		680 (54.4)	415 (62.4)	
Age (years) mean (SD)	37.37 (1.90)	37.46 (1.87)	37.25 (1.93)	.01^d^	37.42 (1.89)	37.26 (1.91)	.06
Race, n (%)				.51			.16
White	1232 (68.5)	699 (69.2)	533 (67.6)		788 (67.5)	436 (70.8)	
Ethnicity, n (%)				.09			.19
Hispanic	179 (9.3)	89 (8.2)	90 (10.6)		108 (8.6)	70 (10.6)	
Marital status, n (%)				.01^d^			<.001^b^
Married	1140 (59.2)	611 (56.7)	529 (62.2)		702 (56.2)	431 (65.0)	
Education, n (%)				<.001^b^			<.001^b^
Some college or below	1132 (58.6)	697 (64.5)	435 (51.1)		807 (64.5)	317 (47.7)	
Living arrangement, n (%)				.21			.003^c^
Own place	1721 (89.3)	953 (88.5)	768 (90.4)		1095 (87.8)	614 (92.3)	
Household income (US $), n (%)				<.001^b^			<.001^b^
<50,000	426 (25.9)	289 (31.3)	137 (18.9)		346 (32.6)	72 (12.6)	
50,000-74,999	286 (17.4)	156 (16.9)	130 (18.0)		186 (17.5)	98 (17.1)	
75,000-99,999	295 (17.9)	161 (17.5)	134 (18.5)		179 (16.9)	115 (20.1)	
100,000-149,999	329 (20.0)	172 (18.7)	157 (21.7)		179 (16.9)	150 (26.2)	
150,000-199,999	164 (10.0)	80 (8.7)	84 (11.6)		93 (8.8)	69 (12.0)	
>200,000	146 (8.9)	64 (6.9)	82 (11.3)		77 (7.3)	69 (12.0)	
Health insurance, n (%)				<.001^b^			<.001^b^
Uninsured	158 (8.2)	117 (10.9)	41 (4.8)		134 (10.8)	22 (3.3)	
Ideology, n (%)				.01^d^			.58
Conservative	521 (28.0)	295 (28.8)	226 (27.0)		339 (28.3)	175 (26.9)	
Middle	837 (45.0)	480 (46.8)	357 (42.7)		541 (45.2)	290 (44.5)	
Liberal	504 (27.1)	250 (24.4)	254 (30.3)		316 (26.4)	186 (28.6)	
Pregnancy, n (%)				.83			.98
Yes	44 (3.0)	26 (3.2)	18 (2.8)		28 (3.0)	16 (3.2)	
Number of children, n (%)				.01^d^			.10
0	106 (7.2)	59 (7.1)	47 (7.4)		74 (7.8)	32 (6.3)	
1	342 (23.3)	172 (20.7)	170 (26.7)		210 (22.2)	131 (25.6)	
2	593 (40.5)	338 (40.7)	255 (40.1)		372 (39.4)	216 (42.3)	
3	285 (19.4)	164 (19.8)	121 (19.0)		184 (19.5)	98 (19.2)	
4	98 (6.7)	65 (7.8)	33 (5.2)		74 (7.8)	23 (4.5)	
5	28 (1.9)	20 (2.4)	8 (1.3)		21 (2.2)	7 (1.4)	
6	14 (1.0)	12 (1.4)	2 (0.3)		10 (1.1)	4 (0.8)	

a Statistical comparisons between users and nonusers were performed using chi-square tests for categorical variables and 1-way ANOVA for continuous variables.

b *P*<.001.

c *P*<.01.

d *P*<.05.

**Table 2.** Different health aspects managed by smartphones (N=851).

	Values, n (%)
Physical activities or routines	714 (84.8)
Diet, food, or calories	399 (47.6)
Weight	359 (42.7)
Heart rate	347 (41.6)
Sleep	276 (33)
Blood pressure	97 (12)
Mood	56 (7)
Medication	54 (7)
Blood sugar or diabetes	24 (3)
Period or menstrual cycle^a^	182 (34.1)
Pregnancy^a^	57 (11)

a Responses were collected from female participants only. Percentage is calculated based on the number of female participants included in the sample (n=534).

**Table 3.** Mean and SD for continuous endogenous variables and correlation matrix (N=1931).

	Mean (SD)	1 (healthy eating)	2 (sleep hours)	3 (physical activity)	4 (BMI)
Healthy eating	66.9 (8.7)	1.00	—^a^	—	—
Sleep hours	6.7 (1.2)	0.12^b^	1.00	—	—
Physical activity	6.5 (5.5)	0.12^b^	0.04	1.00	—
BMI	29.9 (7.2)	−0.10^b^	−0.13^b^	−0.15^b^	1.00
Emotional distress	1.5 (0.5)	−0.15^b^	−0.14^b^	−0.11^b^	0.02

a Not applicable.

b *P*<.001.

### Path Model (H1 and H2)

**Figure 2.**
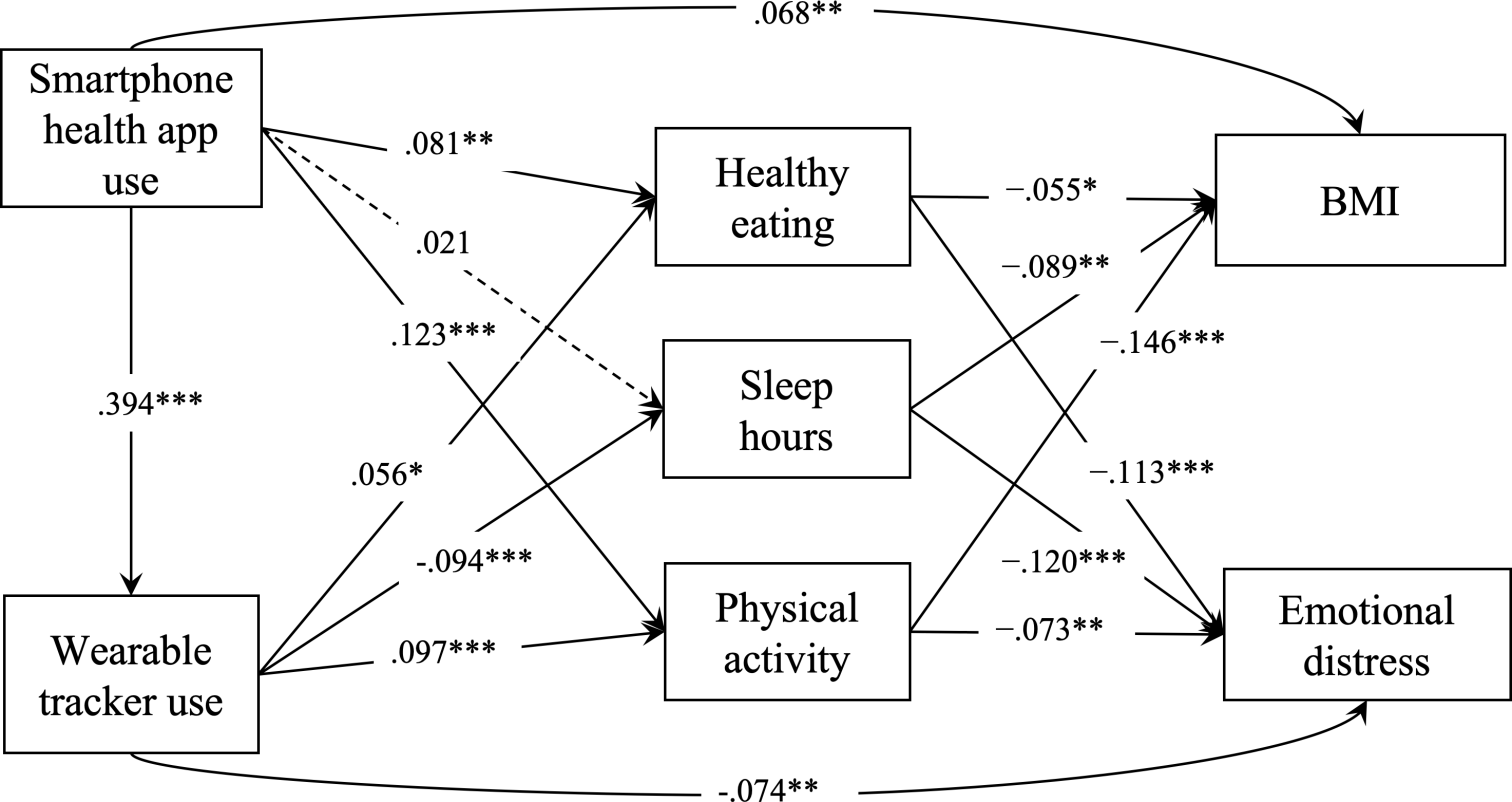
Path model: the relationships among mobile health (smartphone health app use, wearable tracker use), lifestyle mediators (healthy eating, sleep hours, and physical activity), BMI, and emotional distress. Note: solid lines indicate that the path coefficients were significantly different from 0, whereas path coefficients that were not significantly different from 0 were removed for presentation simplicity. Covariates controlled include age, sex, education, household income, race, and ethnicity. Unweighted n=1786. *P<.05, **P<.01, ***P<.001.

The final path model demonstrated a good fit with the data (*χ^2^*_1_=0.9, *P*=.02; root mean square error of approximation=0.048, 90% CI 0.02-0.09; comparative fit index=0.998; standardized root mean square residual=0.005). Results showed that the use of smartphone health apps positively predicted the use of wearable trackers (β=.394; *P*<.001). The use of smartphone health apps predicted higher BMI (β=.068; *P*=.006); thus, H1a was not supported. However, the use of wearable trackers did not significantly predict BMI (β=.047; *P*=.05); thus, H1b was also not supported. Results showed that the use of smartphone health apps did not predict emotional distress (β=−.016; *P*=.49), while the use of wearable trackers significantly predicted lower emotional distress (β=−.074; *P*=.003); thus, H2a was not supported, while H2b was supported. See Figure 2 for the full path model.

### Mediation Analyses (H3 and H4)

Table 4 presents the indirect path coefficients from the mediation analyses to examine H3 and H4. To summarize the pattern, physical activity negatively mediated the associations between both types of mHealth use (health app and wearable tracker) and health outcomes (BMI and emotional distress). Nevertheless, sleep hours positively mediated only the relationship between wearable tracker use and health outcomes. Finally, healthy eating negatively mediated only the associations between both types of mHealth use and emotional distress.

**Table 4.** Indirect path coefficients for mediation analyses.

Indirect path	Estimate	SE	95% CI	β
Smartphone health app → eating → BMI	−0.064	0.034	−0.140 to 0.009	−.004
Smartphone health app → sleep → BMI	−0.027	0.035	−0.105 to 0.037	−.002
Smartphone health app → physical activity → BMI	−0.259	0.068	−0.401 to −0.136	−.018^a^
Wearable tracker → eating → BMI	−0.046	0.027	−0.108 to −0.003	−.003
Wearable tracker → sleep → BMI	0.127	0.051	0.042 to 0.241	.008^b^
Wearable tracker → physical activity → BMI	−0.213	0.068	−0.355 to −0.091	−.014^c^
Smartphone health app → eating → emotional distress	−0.009	0.004	−0.018 to −0.003	−.009^b^
Smartphone health app → sleep → emotional distress	−0.003	0.004	−0.010 to 0.003	−.003
Smartphone health app → physical activity → emotional distress	−0.009	0.004	−0.017 to −0.003	−.009^b^
Wearable tracker → eating → emotional distress	−0.007	0.003	−0.013 to −0.001	−.006^b^
Wearable tracker → sleep → emotional distress	0.012	0.004	0.005 to 0.021	.011^c^
Wearable tracker → physical activity → emotional distress	−0.008	0.004	−0.015 to −0.007	−.007^b^

a *P*<.001.

b *P*<.05.

c *P*<.01.

## Discussion

### Principal Results

This study, based on a nationally representative sample of adults in their late 30s to early 40s, found that mHealth users are more likely to be female, married, and of higher socioeconomic status. These users primarily engage with mobile technologies for tracking physical activity, followed by a range of other personal health aspects. Overall, our findings reveal a nuanced relationship between mHealth use and health outcomes: contrary to expectations, the use of smartphone health apps was associated with higher BMI, and the use of wearable trackers did not significantly predict BMI. However, the use of wearable trackers, not smartphone health apps, was linked to lower emotional distress. Mediation analyses further highlighted the complex mechanisms underlying these associations. Physical activity negatively mediated the relationships between both types of mHealth use and adverse health outcomes. This indicates that mHealth users were more likely to engage in physical activities, which predicted lower BMI and less emotional distress. Sleep hours mediated only the association between wearable tracker use and both health outcomes. This suggests that wearable tracker use was associated with fewer sleep hours, which predicted higher BMI and greater emotional distress. Finally, healthy eating mediated only the relationship between mHealth use and emotional distress. This suggests that mHealth users were more likely to adopt healthy dietary change, which was related to lower emotional distress.

### Comparison With Prior Work

Our findings regarding mHealth use and BMI highlight the mediating role played by lifestyle factors in shaping physical health. Aligned with previous studies, on a positive note, individuals who used smartphones or wearables for health management reported engaging in increased physical activity [9,25], which was associated with lower BMI [17,19,20]. This finding supports the idea that mHealth technologies can potentially serve as effective tools for promoting lifestyle changes that lead to successful weight control. However, while these apps provide valuable information and motivation, their potential effectiveness depends on users taking actionable steps toward improving their health. It is possible that some individuals rely on health apps for tracking or goal setting but struggle with adherence to recommended behaviors, resulting in limited or even counterproductive health outcomes [46]. It is worth noting that the positive direct association between smartphone health app use and BMI can be interpreted in the other way around; people who are of higher BMI tend to use health apps more because they see the need [47]. Future research should explore the psychological and behavioral mechanisms underlying this association.

The relationship between mHealth use and emotional distress also reveals important implications. Using wearable trackers is directly and indirectly associated with lower emotional distress, supporting their potential for mental health intervention [29,31,37]. While smartphone health app use did not show a direct connection to emotional distress, healthy eating and physical activity emerged as key mediators [37-39]. This finding underscores the importance of promoting both a healthy diet and physical activity for emotional well-being rather than relying solely on physical activity [21,33,39]. Future mHealth interventions could enhance their impact by incorporating personalized dietary and workout recommendations to better support emotional well-being.

Finally, an unexpected yet important finding is that wearable tracker use is negatively associated with sleep, which, in turn, is linked to both higher BMI and emotional distress. While our data support the well-established link between sleep and BMI [18], as well as sleep and emotional distress [34,35], they did not provide evidence that mHealth tools designed to improve sleep actually increase the absolute number of sleep hours. This may explain the limited evidence we found regarding the effectiveness of mHealth tools in improving sleep. Even in 1 study that reported wearable tracker use as positively associated with improved sleep, the focus was on perceived sleep quality rather than sleep duration [28]. Additionally, another study cited found that individuals using the wearable tracker had higher levels of well-being before than after its use [40].

There were several speculations on why sleep plays such an alarming mediating role in the relationship between wearable tracker use and health. While wearable devices provide valuable insights into sleep patterns, some users may develop a reliance on these tools, leading to obsessive tendencies where self-worth becomes tied to achieving predefined health metrics, potentially exacerbating emotional distress [37]. Misinterpretation of health data or false alarms may also contribute to heightened anxiety [37], and frequent notifications from these devices can also create a sense of urgency or fear as users may associate them with potential health concerns [48]. Notably, only the use of wearable trackers red-flagged this pattern, while smartphone health app use does not correlate with sleep. This raises the question of whether wearing a physical device itself might cause some sleep disturbances [26]. However, our results do not establish causality; it is also possible that individuals who already experience sleep deprivation and struggle with physical and mental health issues turn to wearable trackers for assistance. Future research is needed to explore these dynamics in greater depth and consider using a tracker to objectively measure sleep or consider sleep quality rather than mere quantity.

### Practical Implications

The findings from this study offer several key implications for the design, development, and implementation of mHealth technologies. First, mHealth designers should prioritize integrating behavioral support features that guide users from self-monitoring to sustained lifestyle changes, given that simply using a smartphone health app without following corresponding lifestyle guidance may be associated with counterproductive outcomes. Health care providers and public health professionals should also avoid an overemphasis on outcome-oriented obsessive-tracking behaviors. Second, mHealth holds promise for mental health intervention, and mHealth solutions could incorporate evidence-based dietary and physical activity programs to further enhance emotional resilience and mental health benefits. Finally, developers should prioritize user-centered design approaches that minimize sleep disruption, such as optimizing comfort, reducing unnecessary notifications, and providing clearer guidance on interpreting sleep data. By addressing these challenges, mHealth technologies can be better optimized to support long-term health benefits while mitigating potential downsides, ultimately improving both physical and emotional well-being among users.

### Limitations

There are several limitations of this study. First, measurement limitations should be acknowledged. For example, the healthy eating measure captures only a subset of dietary behaviors and does not reflect broader aspects of diet quality, the physical activity measure combines frequencies of heterogeneous activities without accounting for intensity, and the sleep variable measures only duration and does not capture quality, disturbances, or restorative aspects of sleep. And the reliance on self-reports may be subject to recall, response, and social desirability biases. Future research could use more comprehensive instruments, include validated indices of behavioral constructs, and incorporate objective health metrics (eg, biometrics from wearables) to enhance data reliability. Second, while this study identifies associations between mHealth use and health outcomes, causal relationships cannot be inferred. Longitudinal studies or experimental designs would provide stronger evidence of causality. Finally, given the growing diversity of mHealth apps, future research should explore how specific app features (eg, gamification and artificial intelligence–driven recommendations) influence user engagement and health behaviors.

### Conclusions

This study underscores the potential of mHealth technologies to improve health outcomes through their influence on lifestyle behaviors. However, their impact is not uniform, and unintended negative consequences such as reduced sleep from wearable tracker use warrant further investigation. By addressing these challenges, mHealth technologies can better fulfill their promise of supporting individuals in achieving healthier lifestyles and advancing population health.

## Abbreviations

mHealth: mobile health
